# Strangulated Gastric Hernia Following a Missed Traumatic Diaphragmatic Injury: A Case Report

**DOI:** 10.7759/cureus.46273

**Published:** 2023-09-30

**Authors:** Maria F Guevara-Kissel, Shamon Gumbs, Javier Andrade, Brian Donaldson

**Affiliations:** 1 Department of Surgery, Columbia University College of Physicians and Surgeons, Harlem Hospital Center, New York, USA; 2 Department of Surgery, NYC Health + Hospitals/Woodhull, New York, USA

**Keywords:** minimally invasive laparoscopy, missed diagnosis, stomach herniation, diaphragmatic rupture, penetrating abdominal trauma

## Abstract

Traumatic diaphragmatic injuries (TDIs) are rare and can be life-threatening, depending on the size of the injury and the contents herniating through it. They usually result from blunt or penetrating trauma to the thoracoabdominal area, with an incidence of 0.8-5% and up to 30% presenting late. A high index of suspicion should be maintained when evaluating patients with a history of trauma (severe blunt or thoracoabdominal penetrating trauma) and upper abdominal symptoms.

We present a case of a missed TDI after a left posterior thoracoabdominal stab injury, which was evaluated with a diagnostic laparoscopy at an outside hospital. He presented to our emergency department (ED) with sudden onset left-sided chest pain and uncontrollable vomiting. A CT scan was obtained and showed a distended stomach herniating through a defect in the left hemidiaphragm. The patient was immediately taken for laparoscopic exploration and repair. There was a 5 cm defect in the left posterolateral diaphragm containing a strangulated stomach (approximately ⅔) and necrotic omentum. Complete reduction was achieved and the diaphragmatic defect was repaired primarily. His postoperative course was uncomplicated. This case illustrates the importance of maintaining a high index of suspicion for TDI, despite reports of previous exploration. Missed TDI can present with herniated intra-abdominal organs, which can become strangulated and increase morbidity and mortality.

## Introduction

Traumatic diaphragmatic injuries (TDIs) are rare, with only a 0.8-5% incidence rate, but up to 30% of cases present late. These injuries can be life-threatening, depending on the size of the rupture and contents that herniate through it, as well as the presence of strangulation or obstruction [1]. Typically, these injuries result from blunt or penetrating trauma to the thoracoabdominal area. While the literature cites an incidence rate of 1-7% for blunt trauma and 10-15% for penetrating injuries [2]. Maintaining a high index of suspicion when evaluating patients with a history of trauma (blunt or penetrating thoracoabdominal) and upper abdominal symptoms is essential. These symptoms may be unspecific, such as chest or abdominal pain, nausea, gastroesophageal reflux disease, or coughing [1,3].

## Case presentation

**Figure 1:**
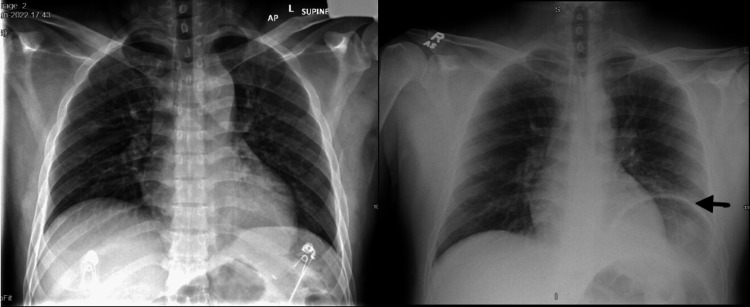
CXR on the left (six months prior) showing no remarkable findings, and CXR on the right showing an elevated left hemidiaphragm (black arrow).

**Figure 2:**
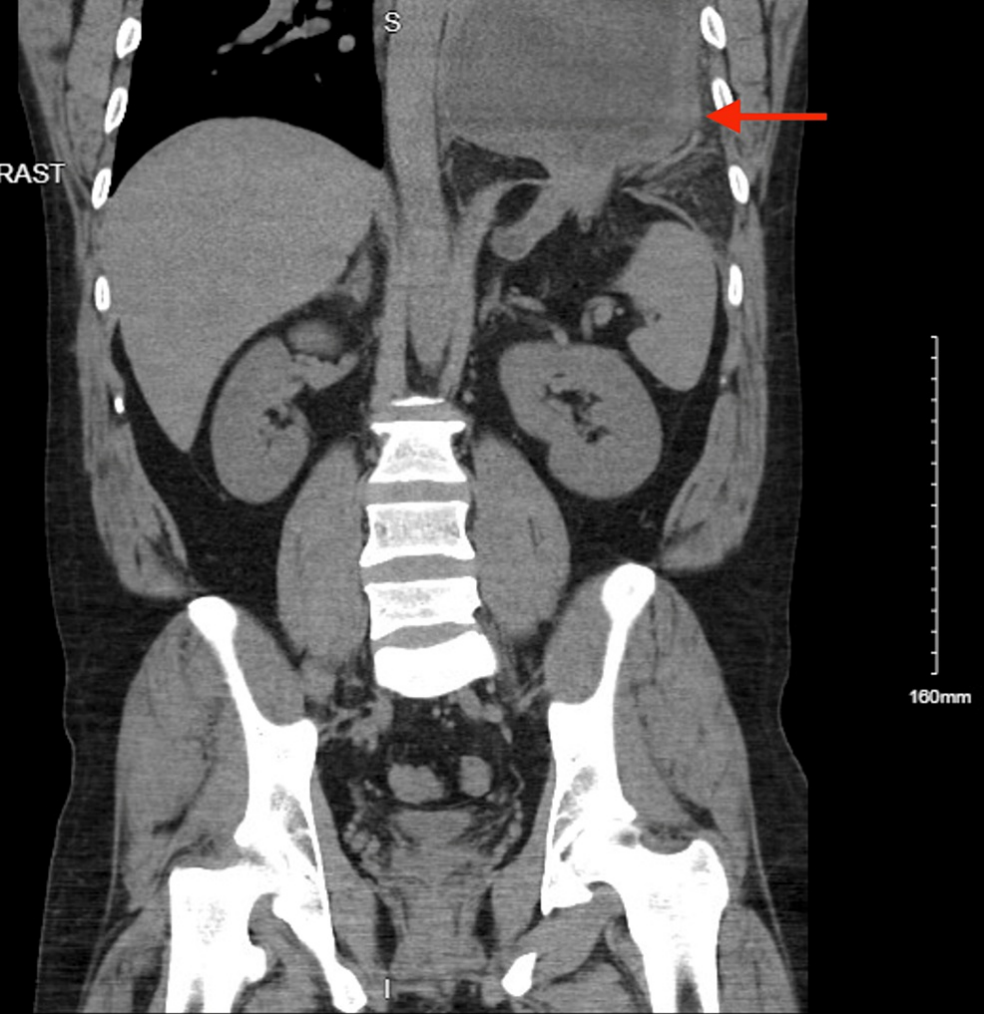
Coronal view on CT of the abdomen/pelvis points to the herniated stomach.

We present a case of a 31-year-old Hispanic male with a one-year history of a left posterior thoracoabdominal stab injury, evaluated with a diagnostic laparoscopy at an outside hospital. He presented to our ED with sudden-onset left-sided chest pain and uncontrollable vomiting. He endorsed chills but no fever. A chest X-ray (CXR) was obtained and reported as unremarkable at that time; however, showed a suspicious elevation in the left hemidiaphragm compared to a previous CXR six months prior (Figure 1). A CT scan was obtained and showed a distended stomach herniating through a defect in the left hemidiaphragm (Figure 2). General surgery was then consulted. Upon surgery evaluation, he was hemodynamically stable, and the abdominal exam was unremarkable. Baseline blood investigation revealed leukocytosis (14.20 x103/mcL), hypokalemia, and elevated lactate (2.5 mmol/L). A nasogastric tube was placed with an immediate return of 700 ml of maroon-colored output. Isotonic crystalloids were given, electrolyte imbalances were corrected, and antibiotic coverage was initiated with Zosyn in preparation for the surgery.

**Figure 3:**
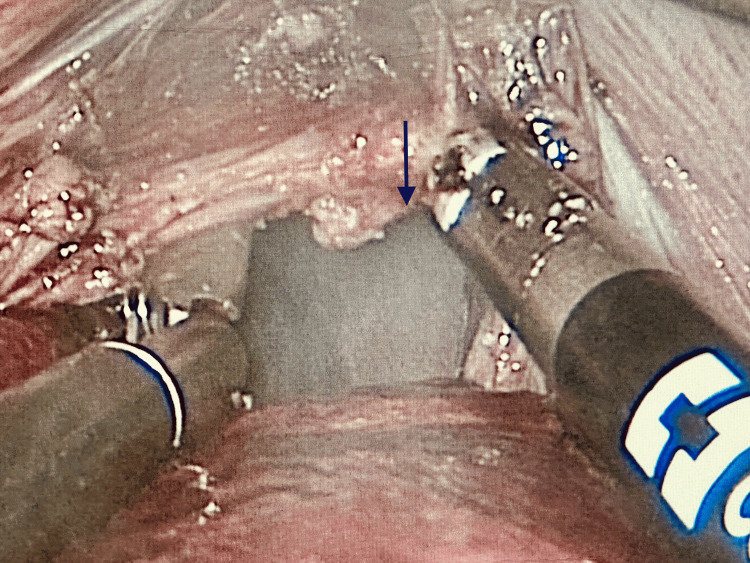
Intraoperative view of the diaphragmatic defect (arrow).

**Figure 4:**
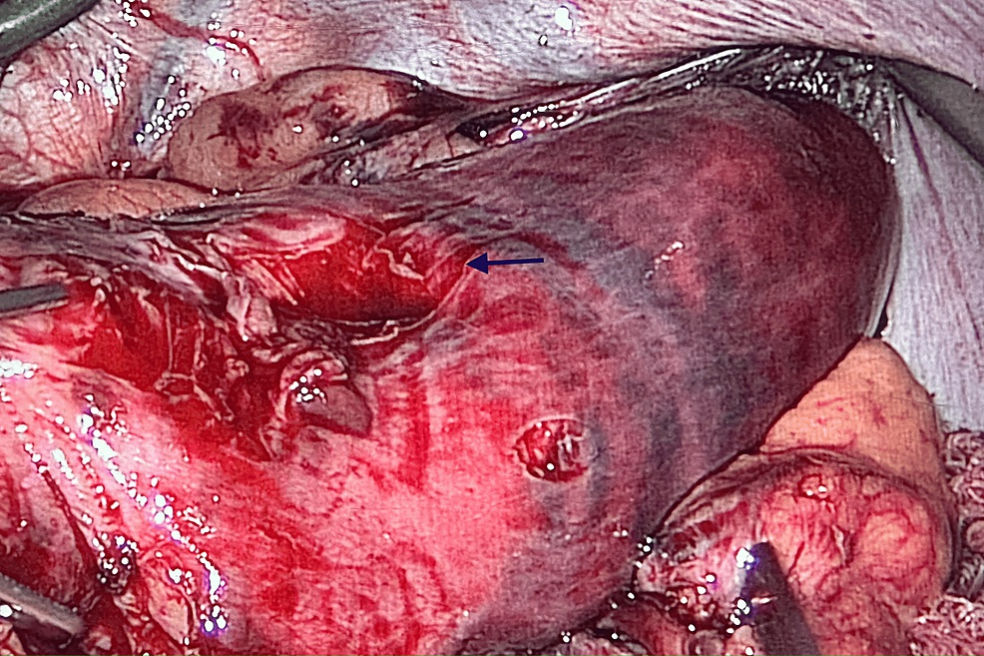
Intraoperative view showing the ischemic changes of the stomach once reduced intra-abdominally and serosal tears (arrow).

Due to the systemic and laboratory signs of developing ischemia in the setting of a herniated stomach with a risk of stomach perforation, the patient was taken for laparoscopic abdominal exploration. There was a 5 cm defect in the left posterolateral diaphragm (Figure 3) containing a strangulated stomach (approximately ⅔ ) and necrotic omentum (Figure 4). The complete reduction was achieved after expanding the defect by 2 cm. The stomach had some serosal tears that were repaired with 2-0 Vicryl Lembert sutures and the necrotic omentum was resected. Left serosanguineous pleural effusion was suctioned and a 32 F chest tube was placed. The diaphragmatic defect was able to be approximated without tension and was repaired primarily using continuous 2-0 V Lock sutures.

His postoperative course was uneventful, he was extubated on postoperative day one, and on postoperative day five, the chest tube was removed after the output was <10 ml in 24 hours for the past 48 hours. He was discharged from the hospital on postoperative day five. During his follow-up visit three weeks after the surgery, he had no issues, and further appointments were scheduled as needed.

## Discussion

TDIs are uncommon and can result from both penetrating and blunt trauma. The reported incidence ranges from 0.8% to 8%, but it is challenging to determine the actual number due to the injuries that may not present until days or even years later, with rupture of the diaphragm and subsequent herniation of abdominal contents [4]. A study by Fair et al. in 2012 analyzing the National Trauma Database Bank identified 3,783 patients with a diagnosis of TDIs, of which 2,543 (67%) were diagnosed with a penetrating diaphragmatic injury, and 1,240 (33%) were diagnosed with a blunt diaphragmatic injury [4]. TDI has been divided into three phases: the acute phase that occurs when the diagnosis is established right after the acute injury or up to 14 days after. The second phase is when it is diagnosed in the period after the acute injury but before obstruction or strangulation occurs [5]. And the third phase occurs when there are complications such as obstruction or strangulation on presentation [5].

A high degree of suspicion is required for proper diagnosis of these injuries as they are often missed even after exploratory laparotomy or laparoscopy. The latter is likely due to the difficulty with direct visualization of both hemi-diaphragms [3]. Also, the fact that penetrating trauma victims tend to have smaller-sized injuries that are not immediately evident [3]. The mechanism of trauma and/or location of the initial injury, as well as associated injuries such as spleen, stomach, liver, or pneumohemothorax, can aid in suspecting a diaphragmatic injury [1].

Delayed presentation is the single most important contributor to increased mortality [6]. This underscores the importance of proper diagnosis and management of this condition.

Diaphragmatic injuries may only be detected after herniation of abdominal contents occurs. The most commonly herniated organs are the stomach, spleen, and bowel. Left-sided ruptures are more common but right-sided ruptures are more difficult to diagnose. The preponderance of left-sided injuries is likely related to the protective effect of the liver on the right side [2].

Diagnosis represents a challenge for clinicians, and in many cases, the patient’s chief complaints are nonspecific, such as chest pain, vomiting, nausea, abdominal pain, general malaise, and breathlessness [7]. Literature has reported that 19% of diaphragmatic ruptures were missed during initial laparotomy [6]. Rupture of devitalized or infected diaphragmatic muscle can occur days after the initial injury, reiterating the importance of maintaining a high index of suspicion in patients with traumatic injuries that can predispose them to a diaphragmatic injury [6]. Imaging plays a vital role in the detection of diaphragmatic defects. A chest radiograph or upper gastrointestinal radiography can represent a preferred method for the initial diagnosis. The diagnostic sensitivity of radiographic imaging such as CT has been historically poor at detecting diaphragmatic injuries initially but its sensitivity increases when organ herniation occurs. Surgical exploration although with the highest sensitivity in detecting injury can be negative in a high percentage of the time [8]. Therefore, because of the low sensitivity in imaging and the significant percentage of surgical explorations that are negative for injury, a high index of suspicion based on the patient’s specific past history is needed to continue with further evaluation that will eventually lead the clinician to the correct diagnosis.

Once the diagnosis of diaphragmatic rupture has been established, management is with surgical reduction of the herniated contents and defect repair. Conservative treatment is not advised as complications involving volvulus, obstruction, or strangulation can arise [9]. Surgical repair has been performed through laparotomy or thoracotomy incisions for many years [10]. However, in more recent years, with advancements in minimally invasive techniques, there has been an increased report of repairs being successful through laparoscopy alone [9-11].

Some of the benefits of the laparoscopic approach include less postoperative pain, faster recovery, and fewer wound complications [10]. The use of prosthetic material such as mesh to close the defect will depend mainly on the size of the defect. Regardless of the approach, it is paramount that the approximation of the edges in the diaphragm defect is done in a tension-free manner [9,10].

It is important to note that TDIs have an overall mortality rate of 21% [12]. Therefore, it is crucial for clinicians working in acute settings to be aware of this entity. When presented with a patient who has suffered from blunt or penetrating trauma, it is important to consider the possibility of diaphragmatic herniation, especially in patients with a past history of thoracoabdominal trauma [13]. By diagnosing these injuries early, we can reduce the morbidity and mortality rates of affected patients.

## Conclusions

TDIs with herniation of abdominal contents are a rare entity; however, they cause significant morbidity and an overall mortality of 21%, or worse when diagnosis is delayed and/or missed. One of the most severe consequences is strangulation followed by sepsis, as was the case with our patient whose systemic and laboratory signs indicated ischemia, later confirmed by intraoperative findings. Therefore, it is crucial for trauma centers to improve their understanding of these injuries to prevent delayed diagnosis. A high index of suspicion should be maintained to potentially diagnose diaphragmatic injuries in patients with the mechanism for a potential injury. If a TDI is diagnosed and repaired on initial presentation, this could avoid later organ herniation through the defect. However, when evaluating patients with a history of blunt or penetrating trauma in the thoracoabdominal region presenting with upper abdominal symptoms, it is key to suspect a late presentation of TDI with herniation of abdominal contents.
